# Sexual Behaviours of Homosexual and Bisexual Men in France: A Generational Approach

**DOI:** 10.1371/journal.pone.0123151

**Published:** 2015-03-27

**Authors:** Nicolas Méthy, Annie Velter, Caroline Semaille, Nathalie Bajos

**Affiliations:** 1 CESP-Inserm U1018, Le Kremlin-Bicêtre, France; 2 Institut de veille sanitaire, Saint-Maurice, France; 3 Agence nationale de sécurité du médicament et des produits de santé, Saint-Denis, France; 4 Institut national d’études démographiques, Paris, France; David Geffen School of Medicine at UCLA, UNITED STATES

## Abstract

**Objective:**

In high-income countries, the social and epidemiological contexts surrounding homosexuality and AIDS have changed profoundly in recent decades. This work sought to examine key indicators of the long-term sexual trajectories of successive generations of men who have sex with men (MSM) in France.

**Methods:**

We performed a longitudinal analysis of the French Gay Press surveys, which were self-administered socio-behavioural questionnaires, repeated from 1985 to 2011 in the gay press, and on the internet in 2004 and 2011. An age-cohort analysis using graphical representations and multivariate logistic regressions was conducted among participants aged 18-59 (N=38 821).

**Results:**

First sexual intercourse occurred more often with a male partner in younger generations than in older ones: 76.0% in MSM who turned 18 in 1956-1959, 75.6% in 1980-1983, 83.7% in 2008-2011, p_overall_=0.0002). Every generation showed the same pattern of sexual trajectory between 1985 and 2011: globally, the frequency of masturbation increased from the 1985 survey to the early 1990s and then decreased from the late 1990s to the end of the study period. Inversely, the frequency of oral and anal sex decreased in the mid-1980s and increased from 1990 to 2011. The frequency of both oral sex and anal intercourse is currently quite high, regardless of generation (>95% and around 80%, respectively). Compared to their predecessors, recent generations of young MSM reported more frequent oral and anal sex, but fewer male partners in the previous 12 months.

**Discussion:**

While the increased frequency of first intercourse with a man over successive generations since the 1970s may be related to reduced social pressure for heterosexuality, there is evidence that sexual norms among MSM are widespread, with practices spreading across age groups and generations. Although AIDS profoundly affected sexual practices in the 1980s, further AIDS-related events (discovery of HIV antiretroviral drugs and their use in prevention) do not appear to have accentuated ongoing trends in sexual practices.

## Introduction

Globally, in both the North and South, regardless of the prevalence of the virus in the general population, the HIV/AIDS epidemic continues to disproportionately affect men who have sex with men (MSM) [1, 2]. In high-income countries, where the HIV epidemic has long been documented, HIV incidence is still high in the MSM population and, contrary to the rate of HIV contamination attributed to heterosexual intercourse, has not decreased [3]. Generational issues are sometimes hypothesised to explain the current HIV situation among MSM, especially young MSM [4–6]. Increases in HIV diagnoses [7], incidence [8] and prevalence [9] in this group have been observed in recent years in some countries, correlated with more high-risk behaviours, which are in turn influenced by various individual and social factors [10]. Understanding how sexual behaviours change over time thus remains essential for exploring the relative contribution of public health policies and secular trends to changes in sexual behaviour and for improving the design of HIV prevention policies [11, 12].

However, analyses of nationwide trends over a long period of time are rare, due especially to the lack of reliable comparable data. Published analyses of MSM have covered various periods and geographical areas (national or specific gay venues, cities or health centres). They have compared cross-sectional surveys [13–21] or HIV-negative and/or HIV-positive MSM cohorts [22–25], but they have not addressed questions of generational differences in a longitudinal approach. Yet, a generational analysis may be of particular interest for several reasons. First, sexuality is a social practice that takes place in given social contexts, from the beginning of sexual life [26–29]; second, the social context concerning homosexuality has changed profoundly in recent decades in high-income countries. Certainly, the sexual behaviours of MSM have long been structured by the negative socio-political environment surrounding homosexuality. Same-sex intercourse was mainly clandestine due to legal and social discrimination. Though stigmatization remains a major concern in some countries around the world [30, 31], MSM in high-income countries have on the whole benefited from legislative progress, enhanced visibility and improved social acceptance since the 1970s. Meanwhile, several generations of MSM have lived through very different AIDS-related contexts: the pre-AIDS era for the oldest generations, whose sexual experience predated the onset of AIDS; the pre-antiretroviral therapy (pre-ART) era, from 1981 to 1995, with many AIDS-related deaths; and the post-ART era since 1996, when antiretroviral drugs became accessible to most people and these treatment improvements progressively converted HIV infection into a chronic disease. Since the late 2000s, the medicalization of prevention has become a topical issue, with antiretrovirals a new tool for combined prevention under the concepts of *Treatment as Prevention* for HIV-positive MSM and *Pre-Exposure Prophylaxis* for HIV-negative MSM [32].

These highly contrasted social and epidemiological contexts might each have had a specific impact on the sexual itineraries of successive generations of MSM, by redefining the context in which same-sex sexuality took place. To our knowledge, however, no large generational analysis retracing sexual trajectories of MSM has yet been published.

This kind of approach has a unique set of supporting documentation in France: the *Enquêtes Presse Gay* (Gay Press Surveys) [33–35], which are repeated nationwide surveys that collected data on how MSM adapted to the HIV/AIDS epidemic from 1985 to 2011. The data permit a retrospective analysis of trends in sexual behaviour over the past six decades, starting at first intercourse, and provide more than 25 years of hindsight concerning sexual practices, from the first signs of the AIDS epidemic up to 2011. Furthermore, the questionnaires are a source of detailed individual information on MSM and their lifestyles, health, sexuality and preventive behaviours.

Here we aim to use this series of French surveys to study the sexual trajectories of successive generations of MSM, from those who started their sexual life before the AIDS epidemic to those who began theirs in the current context of a new HIV prevention paradigm that includes the use of antiretroviral drugs. These trajectories are described in terms of conditions of sexual debut, number of male partners, and sexual practices such as masturbation, oral sex and anal intercourse.

## Materials and Methods

### The Gay Press surveys and their design

The Gay Press surveys are anonymous self-administered socio-behavioural questionnaires (convenience samples) that were repeated from 1985 to 2011 (annually from 1985 to 1993, then in 1995, 1997, 2000, 2004 and 2011). The media recruitment plan changed over time. From 1985 to 1992, a gay magazine with a large nationwide readership (*Gai Pied Hebdo*) included a card containing the questionnaire. When this journal ceased printing, several magazines were included in the survey from 1993 to build up the same variety of profiles [36]: 6 gay magazines in 1993, 10 in 1995, 9 in 1997, 20 in 2000, 16 in 2004 and only one in 2011. In 2004 and 2011, an electronic version of the questionnaire was made available for completion on the internet, accessible via banner ads on various information or meeting websites (10 sites in 2004 and >60 sites in 2011). The different editions were completed by almost 1000 (in 1985 and 1992) to more than 10000 participants (in 2011). Internet accounted for 23% of the questionnaires analysed in 2004 and 90% in 2011. The topics concerned the socio-demographic profile of the respondents, their social lives, their sex lives (practices and prevention methods) and their health.

All questionnaires were completed anonymously. The French data protection committee (*Commission nationale de l'informatique et des libertés*) approved the surveys and data collection and storage.

### Variables for analysis

We compared the data from surveys from which common indicators could be created, restricting analysis to participants aged 18–59 (N = 38821) due to the relatively sparse participation of respondents aged >59. Year of the 18^th^ birthday (= year of birth + 18) was used as the variable defining the cohort/generation, as reference to the starting point of sexual life: 18 was the median age of first sexual intercourse with a man (see Results section) in our samples. This variable allowed us to study MSM who had begun sexual activity (i.e., turned 18) from 1944 to 2011 (1944 corresponding to the oldest MSM included, i.e., those aged 59 in 1985) (Table 1). Other socio-demographic variables were education level, marital status, self-identification and size of the city of residence. We also took into account the source of the questionnaire (press or internet).

**Table 1:** Socio-demographic characteristics of the respondents in the Gay Press surveys.

		1985		1987		1988		1989		1990		1991		1992		1993		1995		1997		2000		2004		2011		Total		p-value
		n	meanage	n	meanage	n	meanage	n	meanage	n	meanage	n	meanage	n	meanage	n	meanage	n	meanage	n	meanage	n	meanage	n	meanage	n	meanage	n	meanage	
Generation (year of 18^th^ birthday)																														
	1944–1947	12	57.6	7	58.4	6	59.0																					25	58.2	
	1948–1951	14	53.4	22	55.1	34	55.9	25	57.5	36	57.9	18	58.6	4	59.0													153	56.7	
	1952–1955	22	49.5	35	51.5	32	52.4	38	53.7	45	54.5	33	55.6	14	57.1	43	57.6	10	58.5									272	54.3	
	1956–1959	33	45.2	30	47.4	53	48.8	55	49.3	54	50.3	51	51.3	26	52.6	49	53.4	23	55.1	28	57.1	11	59.0					413	50.9	
	1960–1963	69	41.0	67	43.6	79	44.4	105	45.2	97	46.3	98	47.3	28	48.1	109	49.3	39	51.3	53	53.2	91	56.1	36	59.0			871	48.1	
	1964–1967	121	37.3	121	39.4	177	40.3	155	41.4	166	42.5	148	43.4	67	44.5	188	45.3	76	47.5	101	49.3	160	52.5	209	56.5			1689	45.4	
	1968–1971	145	33.6	142	35.3	206	36.2	152	37.4	199	38.6	182	39.5	89	40.4	210	41.5	129	43.3	124	45.5	194	48.5	243	52.5	127	58.5	2142	42.5	
	1972–1975	177	29.4	176	31.4	201	32.5	203	33.5	280	34.4	258	35.4	106	36.6	350	37.3	206	39.4	256	41.4	306	44.4	317	48.5	412	55.4	3248	40.1	
	1976–1979	179	25.4	210	27.4	231	28.5	246	29.3	292	30.4	327	31.4	149	32.4	538	33.4	334	35.3	369	37.4	523	40.4	570	44.4	633	51.4	4601	37.1	
	1980–1983	129	21.6	211	23.5	208	24.7	256	25.5	336	26.4	336	27.5	150	28.5	659	29.5	514	31.4	575	33.4	725	36.4	753	40.4	970	47.4	5822	34.2	
	1984–1987	31	18.6	109	19.9	105	21.0	158	21.9	232	22.8	216	23.6	103	24.8	590	25.6	557	27.5	594	29.5	824	32.5	834	36.5	1197	43.5	5550	32.2	
	1988–1991					13	18.0	32	18.7	56	19.2	90	20.1	56	21.1	346	21.9	500	23.6	656	25.6	814	28.5	894	32.5	1272	39.5	4729	30.4	
	1992–1995													3	18.0	43	18.7	157	20.0	375	21.7	545	24.7	754	28.6	1115	35.6	2992	29.0	
	1996–1999																			74	18.6	322	20.7	624	24.6	1136	31.5	2156	27.5	
	2000–2003																					35	18.0	413	20.6	1232	27.5	1680	25.6	
	2004–2007																							47	18.0	1407	23.6	1454	23.4	
	2008–2011																									1024	19.7	1024	19.7	
	Total	932	31.5*8*.*39* ^*a*^	1130	31.8*8*.*99* ^*a*^	1345	33.7*8*.*93* ^*a*^	1425	33.4*9*.*19* ^*a*^	1793	33.6*9*.*19* ^*a*^	1757	33.7*8*.*85* ^*a*^	795	34.1*8*.*76* ^*a*^	3125	32.5*8*.*41* ^*a*^	2545	31.2*7*.*79* ^*a*^	3205	32.0*8*.*22* ^*a*^	4550	34.2*8*.*77* ^*a*^	5694	35.8*9*.*64* ^*a*^	10525	35.5*10*.*6* ^*a*^	38821	34.0*9*.*48* ^*a*^	*p*<0.0001
		**n**	**%**	**n**	**%**	**n**	**%**	**n**	**%**	**n**	**%**	**n**	**%**	**n**	**%**	**n**	**%**	**n**	**%**	**n**	**%**	**n**	**%**	**n**	**%**	**n**	**%**	**n**	**%**	
Education level																														*p*<0.0001
	< baccalaureate	249	27.1	291	25.8	382	28.5	351	24.9	481	27.0	431	24.6	183	23.1	727	23.3	501	19.8	631	19.7	727	16.1	1001	17.7	1633	15.7	7588	19.7	
	Bac/ university degree	319	34.7	494	43.8	506	37.8	545	38.6	647	36.3	666	38.1	269	34.0	1183	38.0	976	38.5	1241	38.8	1669	37.0	2222	39.2	3853	37.0	14590	37.8	
	Post graduate/ PhD	352	38.3	343	30.4	452	33.7	516	36.5	652	36.6	652	37.3	339	42.9	1205	38.7	1059	41.8	1327	41.5	2119	46.9	2444	43.1	4926	47.3	16386	42.5	
	Total	920	100	1128	100	1340	100	1412	100	1780	100	1749	100	791	100	3115	100	2536	100	3199	100	4515	100	5667	100	10412	100	38564	100	
Marital status																														*p*<0.0001
	Single	806	86.6	993	87.9	1171	87.1	1253	88.3	1550	86.5	1564	89.1	696	87.5	2800	89.7	2264	89.0	2955	92.4	4127	92.2	5167	92.5	9574	93.5	34920	91.1	
	Married, divorced or widowed	125	13.4	137	12.1	173	12.9	166	11.7	242	13.5	192	10.9	99	12.5	322	10.3	279	11.0	244	7.6	351	7.8	418	7.5	669	6.5	3417	8.9	
	Total	931	100	1130	100	1344	100	1419	100	1792	100	1756	100	795	100	3122	100	2543	100	3199	100	4478	100	5585	100	10243	100	38337	100	
Place of residence																														*p*<0.0001
	City ≤100000 inhabitants	554^b^	59.7^b^	372	33.2	426	32.2	425	30.0	483	27.2	621	36.8	188	23.8	762	24.5	708	28.0	867	27.7	1248	27.9	1842	32.9	3857	36.7	11799	31.5	
	City >100000 inhabitants			306	27.3	328	24.8	340	24.0	415	23.4	465	27.5	207	26.2	725	23.3	626	24.8	878	28.0	1315	29.3	1839	32.8	3229	30.7	10673	28.5	
	Paris region	374	40.3	444	39.6	568	43.0	651	46.0	876	49.4	602	35.7	394	49.9	1618	52.1	1191	47.2	1390	44.3	1918	42.8	1923	34.3	3424	32.6	14999	40.0	
	Total	928	100	1122	100	1322	100	1416	100	1774	100	1688	100	789	100	3105	100	2525	100	3135	100	4481	100	5604	100	10510	100	37471	100	
Self-identification																														*p*<0.0001
	Homosexual/gay	678	73.9					1139	81.4			1427	81.3			2639	84.6	2211	87.1	2820	88.5	4119	91.2	5041	90.1	9190	87.8	29264	87.4	
	Bi or heterosexual	103	11.2					144	10.3			146	8.3			310	9.9	222	8.7	182	5.7	196	4.3	340	6.1	1045	10.0	2688	8.0	
	Refuses to identify	137	14.9					117	8.4			182	10.4			172	5.5	105	4.1	184	5.8	202	4.5	211	3.8	232	2.2	1542	4.6	
	Total	918	100					1400	100			1755	100			3121	100	2538	100	3186	100	4517	100	5592	100	10467	100	33494	100	

Respondents aged 18–59.

^a^ Standard deviation

^b^ ≤100000 and >100000 confounded

The sexual debut was described by the sex of the first sexual partner and the age at the first intercourse with a man, from data in surveys from 1995 to 2011. First intercourse is used here to translation for *premier rapport sexuel* in French. No definition was provided in the surveys; it refers to first sexual experience. Individuals interviewed with this question and then asked about their interpretation of the questions generally understood the term as indicating penetrative sexual relations [37]. Questions used for the analysis of sexual debut were: *“How old were you when you had your first sexual intercourse with a man*?*”* and *“How old were you when you had your first sexual intercourse with a woman*?*”*. When reported age was the same for both, the sex of the very first partner could not be determined and was considered as missing. This concerned only 4.6% (723/15759) of the respondents of the 1995–2004 surveys. In 2011, a supplementary question provided this information.

We examined the percentages of men who had more than 10 male partners in the previous six months from 1985 to 1990 and in the previous 12 months from 1991 to 2011. We also analysed the rates at which reciprocal masturbation, oral sex and anal intercourse were reported as frequent practices with any partner (steady or casual) from 1985 to 2011. The practice was coded frequent if the respondent answered “always” or “often” (vs. “sometimes” and “never”). Questions referred to different recall periods according to survey year: i) no recall period before 1989; ii) recall period of six months for 1989–1990 and iii) 12 months after 1990.

### A preliminary analysis: Comparability of the samples

The Gay Press surveys are convenience samples obtained from varying sources. Trends observed can thus result from real changes in sexual behaviours or from changes in the socio-demographic structure of the respondents. One way to verify the comparability of the samples is to test if indicators that are not supposed to change over time within a given generation are actually stable. For instance, age at first intercourse reported by MSM of the 1980–1983 generation should be the same in the 1995 survey when they are 30–33 years old as in the 2004 survey when their age is 39–42. This means that, after controlling for generation, no significant effect of the survey year should remain. We used linear and logistic models to test this assumption for education level (higher education in respondents aged ≥ 20), sex of the first partner and age at first intercourse with a man; survey years were included as dummy variables. Models were also adjusted for age at survey to take into account the participation bias linked to age in this sort of convenience survey [38, 39]. Age and generation were included in the models as continuous variables after fractional polynomial transformation when it improved model fit [40].

Results of this preliminary analysis showed that while the survey year had a highly significant effect (*p*_year_<0.0001) on whether subjects had at least a university degree in a bivariate logistic regression including age, it was no longer significant after further adjustment for generation (*p*_year_ = 0.20). No year effects remained significant concerning first sexual intercourse with a man (*p*_year_ = 0.80) or on age at that event (*p* = 0.93). These results support the stability of the reporting at the generational level and the comparability of the samples.

### Statistical analysis

The categorical variables were reported as percentages, and distributions were compared with Pearson’s Chi-squared test. We tested linear trends in bivariate logistic models with the survey year included as a continuous variable. Concerning quantitative variables, mean age differences between surveys were assessed with a bivariate linear model in which the survey year variable was included as a categorical variable. The median age at first intercourse with a man was estimated by linear interpolation.

**Fig 1:**
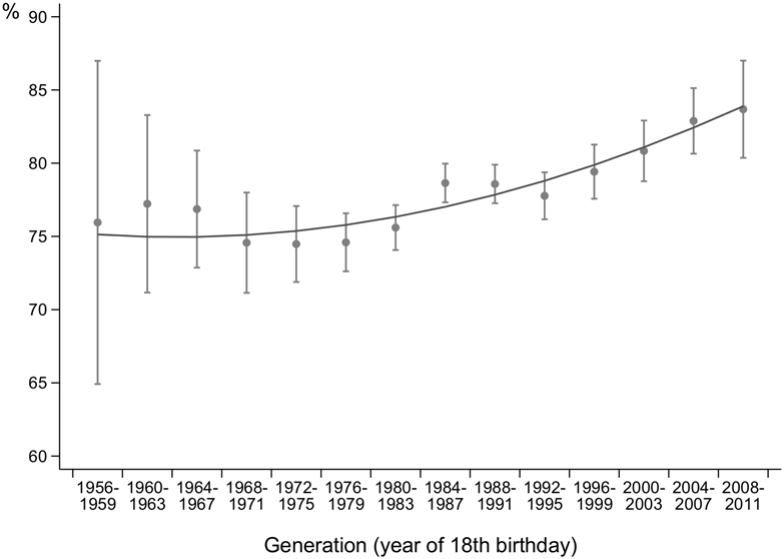
Percentages of respondents reporting that their first sexual intercourse was with a male partner, adjusted for age, and corresponding fit curve. Surveys in 1995–2011, respondents aged 18–59.

**Fig 2:**
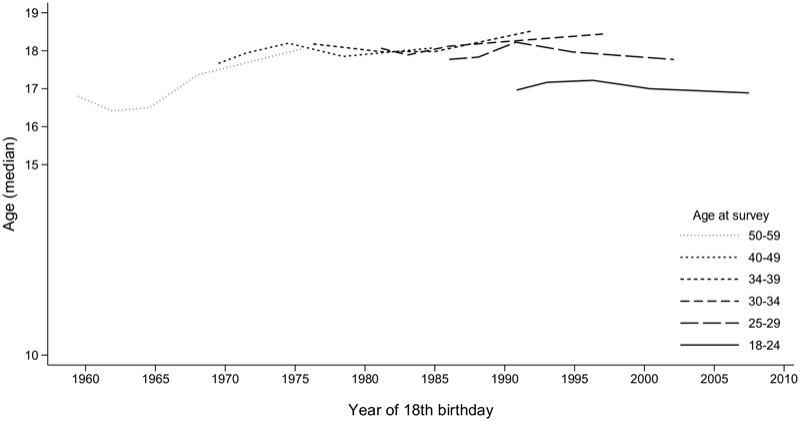
Median age at first sexual intercourse with a man. Surveys in 1995–2011, respondents aged 18–59. Reading: Respondents aged 25–29 surveyed in 1995 had turned 18 in 1986 on average and had a median age at first intercourse with a man equal to 17.8. Lines are drawn only when the generation is composed of at least 30 respondents.

**Fig 3:**
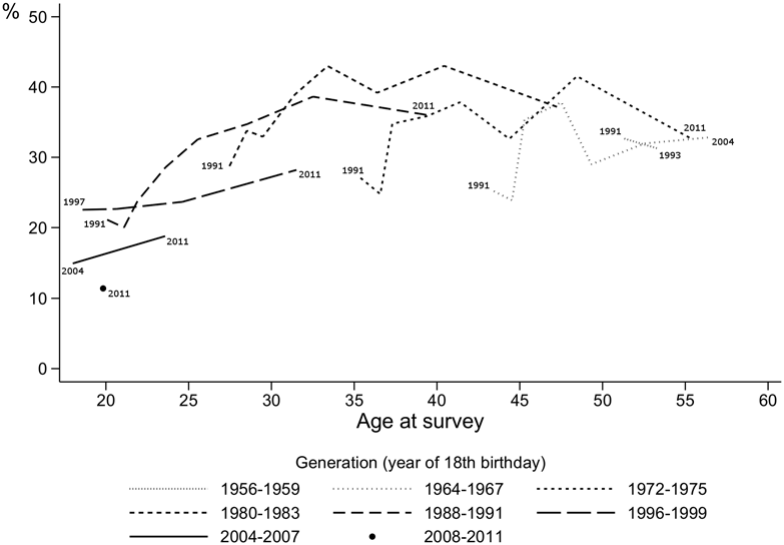
Percentage of participants reporting more than 10 male partners in the previous 12 months. Surveys in 1991–2011, respondents aged 18–59. Reading: 22.5% of the respondents from the 1996–1999 generation reported more than 10 male partners in the previous 12 months when their mean age was 18.6 years (i.e., when they were surveyed in 1997). Lines are drawn only when the generation is composed of at least 30 respondents.

**Fig 4:**
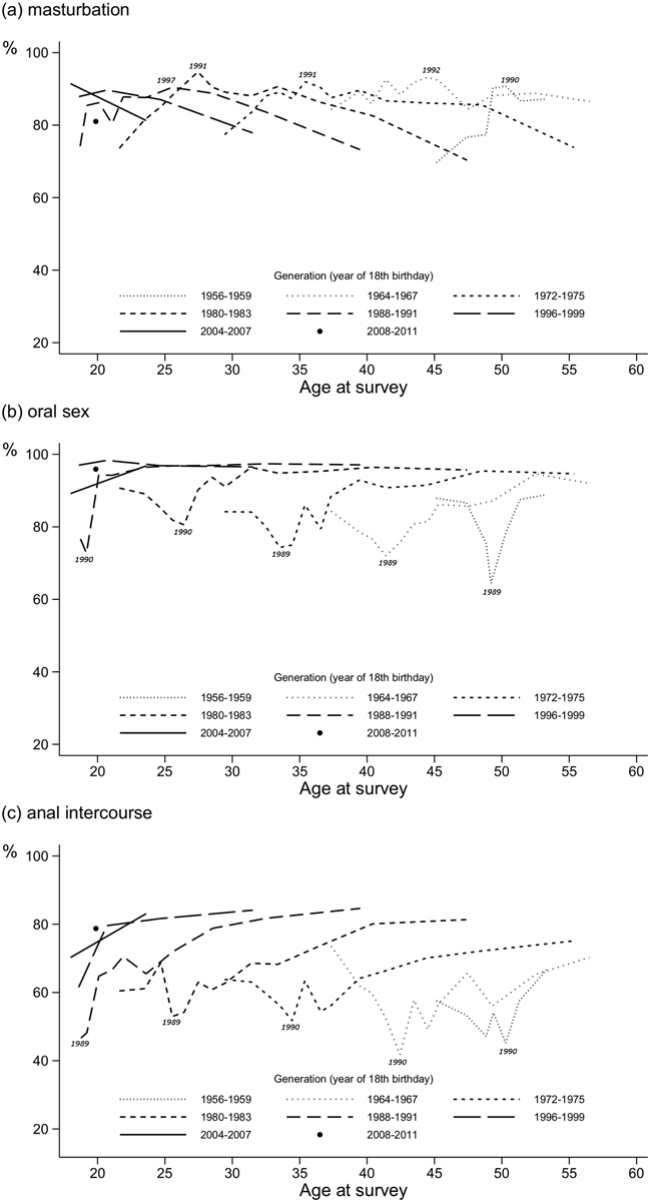
Percentage of participants reporting (a) masturbation, (b) oral sex, and (c) anal intercourse as frequent practices. Surveys in 1985–2011, respondents aged 18–59. Reading: 61.5% of the respondents from the 1996–1999 generation reported frequent anal intercourse when their mean aged was 18.6 years (i.e., when they were surveyed in 1997).Lines are drawn only when the generation is composed of at least 30 respondents.

Graphical representations were used to compare sexual itineraries of the successive generations. To take age-related participation bias (see above) into account, percentages of first intercourse with a man by generations were calculated from an age-adjusted logistic model (Fig 1), and median age at first intercourse with a man was represented by age group (Fig 2). Standard age-cohort representations on raw data were used for the number of male partners (Fig 3) and sexual practices (Fig 4).

Finally, a multivariate logistic regression was conducted to examine factors associated with reporting more than 10 partners in the previous 12 months from the 1991 to the 2011 surveys. Age and age squared were included in the model to adjust for the quadratic effect of age on the dependent variable. We used the Wald test to check for interactions between each of the confounding variables (questionnaire source, self-defined sexual orientation, size of city of residence and level of education) and age (linear term) and generation.

Stata 13 was used to manage the data of the 13 available surveys and statistical analyses.

## Results

### Profiles of MSM in the surveys (Table 1)

The mean age of the respondents varied between 31.2 and 35.8 years from one survey to another (*p*<0.0001). The percentage of participants with a high level of education increased steadily from 1985 to 2011 (linear trend: *p*<0.0001).

The proportion of participants currently or ever married decreased over time (from 13.3% in 1985 to 6.4% in 2011) (linear trend: *p*<0.0001). The proportion of respondents living in Paris or the Paris region was significantly lower in 2004/2011, when the media plan included the internet, than in 1985–2000 (45.2% vs. 33.2%, *p*<0.0001).

The vast majority of respondents defined themselves as homosexual or gay. This percentage rose between 1985 and 2000, from 73.9% to 91.2%, respectively (linear trend: *p*<0.0001), while the proportion of bi and heterosexuals decreased (linear trend: *p*<0.0001). The 2004 and 2011 surveys recruited more bi and heterosexuals than previously, with a higher proportion in the internet samples than in the press samples: in 2004, 6.1% overall, 4.8% from the press, 10.5% from the internet (*p*<0.0001); in 2011, 10.0% overall, 4.1% from the press, 10.6% from the internet (*p*<0.0001). The proportion of participants who refused to define themselves decreased throughout the 1985–2011 period (linear trend: *p*<0.0001).

### Sexual debut

Fig 1 represents the age-adjusted percentages of respondents who reported that their first sexual intercourse was with a man, by generation. It shows that younger generations had their first intercourse with a man more frequently than the older ones did: 76.0% in the 1956–1959 generation, 75.6% in the 1980–1983 generation, and 83.7% in the 2008–2011 generation (p_overall_ = 0.0002).

Fig 2 shows median age at first sexual intercourse with a man according to year of 18^th^ birthday. The median age at this event remained stable at around 18, beginning with the 1970 generation. As explained above, younger respondents (aged 18–24) had a lower median age at first intercourse (around 17) due to participation bias; after adjustment for age, age at first sex did not differ between the 1968–1971 generations and those that followed them (*p* = 0.21).

### Number of male partners

The percentage of respondents reporting more than 10 male partners in the previous six months decreased between the 1985 and 1987 surveys: 27.2% in 1985, 15.7% in 1987, then 17.1% in 1988 and 17.0% in 1989. Similar trends were observed in every generation (data not shown).

Fig 3 shows that age had a global effect on the number of sexual partners: those at the beginning of sexual life had more than 10 male partners less often than older men (30 or older). A difference between generations also existed: comparison of the curves shows that, at a given age, from the oldest to the 1980–1983 generation, the number of sexual partners increased in each successive generation (i.e., the curve is higher for each newer generation). After the 1980–83 generation, however, this trend tended to reverse: recent generations of young MSM reported fewer male partners than older generations at the same age.

The multivariate analysis of data from the 1991–2011 surveys (Table 2) confirmed a generational trend. The odds of having had more than 10 male partners in the previous 12 months increased from the oldest generation to the early 1980s generations (compared to the 1980–1983 generation, aOR = 0.73 [0.56,0.94], *p* = 0.017 for the 1960–1963 generation and aOR = 0.80 [0.71,0.91] *p*<0.0001 for the 1972–1975 generation). It then decreased for the more recent generations (aOR = 0.81 [0.69,0.94], *p* = 0.006 for the 1996–1999 generation; aOR = 0.52 [0.37,0.73], *p*<0.0001 for the 2008–2011 generation). Regardless of the generation, self-defined bi or heterosexual MSM and MSM who refused to identify reported significantly fewer male partners than homosexual/gay MSM: aOR = 0.65 [0.59,0.72], *p*<0.0001 compared with aOR = 0.82 [0.72, 0.93], *p* = 0.002, respectively. Living in a city of over 100000 inhabitants and living in the Paris region were associated with an increased probability of reporting more than 10 male partners in the previous 12 months compared to living in cities with a population under 100000: aOR = 1.44 [1.34,1.54], *p*<0.0001 and aOR = 1.74 [1.64, 1.86], p<0.0001, respectively. The positive association between high educational level and number of partners increased with age: aOR post-graduate/PhD vs. <baccalaureate = 0.97 [0.87,1.09], *p* = 0.64 for MSM aged 25 and 1.51 [1.28,1.77], *p*<0.0001, for those aged 55. Recruitment by internet (compared with by press) was negatively associated with more than 10 partners only at younger ages: aOR = 0.85 [0.73,0.99], *p* = 0.035 and aOR = 1.09 [0.92,1.29], *p* = 0.30, for the same two age groups.

**Table 2:** Factors associated with reporting more than 10 male partners in the previous 12 months.

			raw % of participants with > 10 male partners	adjusted OR [95% CI]	*p*-value
**Age**					<0.0001
**Age^2^**					<0.0001
**Generation (year of 18** ^**th**^ **birthday)**					<0.0001
		1948–1951	22.2	0.50 [0.16,1.59]	0.24
		1952–1955	24.7	0.62 [0.36,1.07]	0.085
		1956–1959	30.7	0.78 [0.52,1.15]	0.21
		1960–1963	31.6	0.73 [0.56,0.94]	0.017
		1964–1967	31.8	0.71 [0.58,0.86]	0.001
		1968–1971	33.1	0.75 [0.64,0.88]	<0.0001
		1972–1975	34.9	0.80 [0.71,0.91]	<0.0001
		1976–1979	38.5	0.96 [0.87,1.06]	0.39
		1980–1983	38.2	1	
		1984–1987	36.5	1.01 [0.92,1.10]	0.83
		1988–1991	33.7	0.99 [0.90,1.10]	0.90
		1992–1995	31.6	0.98 [0.87,1.10]	0.74
		1996–1999	25.8	0.81 [0.69,0.94]	0.006
		2000–2003	21.9	0.76 [0.62,0.92]	0.005
		2004–2007	18.7	0.73 [0.57,0.93]	0.011
		2008–2011	11.1	0.52 [0.37,0.73]	<0.0001
**Self-identification**					<0.0001
		Homosexual/gay	33.5	1	.
		Bi or heterosexual	23.0	0.65 [0.59,0.72]	<0.0001
		Refuses to identify	29.1	0.82 [0.72,0.93]	0.002
**Place of residence**					<0.0001
		City ≤100000 inhabitants	25.1	1	.
		City >100000 inhabitants	32.5	1.44 [1.34,1.54]	<0.0001
		Paris region	38.5	1.74 [1.64,1.86]	<0.0001
**Education level**					<0.0001^a^
	*at 25 years old*	< baccalaureate	27.1	1	
		Bac/ university degree	26.1	1.09 [0.98,1.22]	0.13
		Post graduate/ PhD	25.1	0.97 [0.87,1.09]	0.64
	*at 35 years old*	< baccalaureate	28.6	1	
		Bac/ university degree	36.9	1.12 [1.04,1.21]	0.002
		Post graduate/ PhD	38.1	1.13 [1.05,1.21]	0.002
	*at 45 years old*	< baccalaureate	31.1	1	
		Bac/ university degree	40.3	1.16 [1.05,1.28]	0.005
		Post graduate/ PhD	40.6	1.30 [1.18,1.44]	0.000
	*at 55 years old*	< baccalaureate	28.4	1	
		Bac/ university degree	27.4	1.19 [1.01,1.41]	0.036
		Post graduate/ PhD	40.8	1.51 [1.28,1.77]	<0.0001
**Source of questionnaire**					0.068^a^
	*at 25 years old*	Press	28.5	1	
		Internet	19.8	0.85 [0.73,0.99]	0.035
	*at 35 years old*	Press	36.4	1	
		Internet	35.2	0.92 [0.84,1.01]	0.093
	*at 45 years old*	Press	36.0	1	
		Internet	41.6	1.00 [0.91,1.11]	0.93
	*at 55 years old*	Press	29.4	1	
		Internet	38.1	1.09 [0.92,1.29]	0.30

Surveys in 1991–2011, respondents aged 18–59, N = 29790

^a^ *p* for interaction with age

### Sexual practices

#### Same patterns for each generation

Fig 4 shows the frequency trends for (a) reciprocal masturbation, (b) oral sex and (c) anal intercourse between 1985 and 2011 by age per generation. The same trajectory was observed for each sexual practice in every generation: regardless of age and generation, (a) frequent masturbation increased from 1985 to the early 1990s and then decreased until 2011 (e.g., for the 1980–1983 generation: 73.6% in 1985 (mean age = 21.6 years), 94.7% in 1991 (mean age = 27.5 years), 70.3% in 2011 (mean age = 47.4 years)); (b) frequent oral sex decreased from 1985 to 1989/1990 and then increased until the 2000s and stabilised at a high level (e.g. for the 1980–1983 generation: 90.7% in 1985, 80.6% in 1990, 95.7% in 2011); (c) frequent anal sex decreased from 1985 to 1989/1990 and then increased until 2011 (e.g. for the 1980–1983 generation: 60.5% in 1985, 53.0% in 1989 and 81.4% in 2011).

The most marked change in these trajectories occurred around the year 1990, for masturbation as well as for oral sex and anal intercourse. The corresponding trends are, however, reversed between masturbation, on the one hand, and oral sex and anal intercourse on the other: as frequent masturbation increased in the 1980s, frequent oral sex and anal intercourse decreased; and as frequent masturbation decreased in the 1990s and 2000s, frequent oral sex and anal intercourse increased.

The change in the recall period might have played a role, although it is hard to argue that the frequency of a practice is likely to differ much “in the past six months” versus “in the past 12 months”. In any case, even if we excluded the 1989 and 1990 surveys, which included the “past six months” questions, these trends would still be present.

#### Increases in oral sex and anal intercourse in each successive generation

At any given age, the most marked differences between generations concerned anal intercourse, and, to a lesser extent, oral sex. Their frequencies increased in each successive generation. For instance, at around 32 years old, frequent anal intercourse (Fig 4c) was reported by 60.3% of the MSM of the 1972–1975 generation, 68.6% of the 1980–1983 generation, 81.7% of the 1988–1991 generation, and 84.1% of the 1996–1999 generation. Contrary to our observations about the number of sexual partners, for which the younger generations reported fewer partners than older ones, MSM of the generations ≥1996–1999 reported frequent oral sex and anal intercourse more often than older generations at the same age. Frequent oral sex has reached a very high level (> 95%) and frequent anal intercourse a high level (about 80%) today. They are both reported by MSM even at the beginning of their sexual life.

## Discussion

In our study, since the 1970s, the more recent the generation of MSM, the more often their first sexual experience was with a man. Age at first sexual intercourse with a man has nonetheless remained stable, with a median age of about 18. Every generation showed the same pattern of sexual trajectory between 1985 and 2011: while masturbation increased from 1985 to the early 1990s and decreased thereafter, the number of male partners and the frequency of oral and anal sex decreased in the mid-1980s and have increased since 1990. Recent generations of respondents were more likely to report frequent oral sex and frequent anal intercourse than older ones, but also fewer male partners in the past 12 months.

As community-based convenience samples, these surveys are not representative of the French MSM population [41, 42]. A non-probability sampling scheme is however used most often to survey MSM because they are a so-called “hard-to-reach” population and they represent too small a percentage of the general population to be recruited in nationally representative surveys [43]. Nonetheless, comparison of a series of convenience surveys remains possible as long as there are no differential biases between samples. In our study, comparability is strengthened by the intra-generational stability over time of some indicators concerning past events (university degree, sex of first partner and age at first intercourse with a man). The larger proportion of bi and heterosexual men in recent surveys (mainly in 2011) is likely due to the greater propensity of bisexuals today to answer this kind of survey because they are closer to the media that relay it (internet in particular) rather than to a strong change in sexual behaviours.

Furthermore, convenience sampling methods are known to induce participation bias linked to age [38, 39]. This is a major issue in this work for two reasons: first, self-selection of participants according to their age is particularly obvious in gay surveys: participating in such surveys involves buying a gay journal or spending time at gay websites, and answering questions about sexual behaviours. Younger participants are likely to be the most self-assured of their generation about their own homosexuality. This point is illustrated by the finding that the youngest participants (18–24) enrolled in the surveys had a median age at first intercourse with a man lower than that of older participants. We may also suppose that older MSM who continued to participate in the surveys were the most involved in sexuality and/or invested in gay life. The second reason is that we conducted an age-generation analysis, for which knowledge of the age effect is essential for interpreting the sexual trajectories of the successive generations of MSM. Accordingly, we used bi- and multivariate models that allowed us to adjust for age in addition to other potential confounding factors, such as educational level, size of city of residence, self-defined sexual orientation and source of the questionnaire.

Even though these methodological issues require that our results be interpreted cautiously, this work presents an original longitudinal analysis that offers a new perspective on sexual behaviour trends.

Our results describe changes in sexual itineraries of MSM associated with trends in the social context surrounding homosexuality. An earlier study of the Gay Press surveys, from 1985 to 1995, showed that MSM who had their first sexual experience before 1970 (i.e., before the emergence of social movements for the recognition of marginal sexualities) were more likely to have lived a heterosexual life (for example, had married) than more recent generations [44]. Our finding that since the 1970s the first sexual experience has progressively occurred more and more frequently with a man shows that improved social acceptance of homosexuality and lesser social pressure for heterosexuality have also translated into sexual behaviours from the beginning of the sexual life. In France, the decriminalisation of homosexuality in 1982, the legal recognition of same sex couples in 1999 [45] and civil marriage in 2013 are all major legislative steps in the progress of sexual minorities. They followed the social movements of the 1970s and the gay mobilization and advocacy that has accompanied the increase in social acceptance since the mid-1980s. Nevertheless, this more favourable social context has not eliminated the difficulties that some MSM still experience in living with their homosexuality [30, 46–48].

Our results also show evidence of widespread community norms in sexual practices. The trends observed in the pre-ART era for number of sexual partners and sexual practices were the same for all generations, regardless of age. AIDS had a deep impact on the sexual practices of all MSM, with a drop in the number of male partners in the 1980s, less frequent oral sex and anal intercourse, and more frequent masturbation. This can be seen as a safety reaction during a period of uncertainty and lack of scientific knowledge about AIDS. These changes in sexual behaviour were in accordance with the first community-based preventive interventions conducted in France by gay associations in the mid-1980s to inform MSM about AIDS [49]. In 1987, the first national campaign directed at the general population promoted the use of condoms [50]. Meanwhile, studies agreed that the risk associated with oral sex was low. Thus, MSM adopted condoms for anal intercourse and reengaged in penetrative practices, with an increasing number of partners in the 1990s. This occurred during a period of AIDS mortality rates that remained high (the number of AIDS deaths in France peaked in 1994 [51]).

Similar trends in the frequency of sexual practices were also observed among all generations in the post-ART era. In particular, we observed no differences during that period between MSM who experienced the dark years of AIDS in the 1980s and 1990s and those with sexual debuts after the efficacy of antiretroviral drugs was demonstrated in 1996. Practices seem to have spread in the community across age groups and through successive generations, although the community landscape had been changing profoundly for several decades and the “gay community” is a complex and protean concept [52]. Social community norms in terms of sexual practices remain strong, regardless of the development of new areas of sexual socialization, such as the internet [53].

Although the youngest generations (≥ 1996–1999) reported more frequent oral sex and anal intercourse than older ones at the same age, they reported more than 10 male partners in the past 12 months less often. This generational trend remained after adjustment for self-identification and source of questionnaire. This result seems quite concordant with the decrease in the number of partners observed in the US since the beginning of the 2000s [16, 20] and may reflect differential evolutions in sexual networks, which may mostly concern the young generations of MSM.

These trends also revealed that epidemiological events and preventive concerns have not necessarily had an immediate and sustainable impact on sexual behaviours. Contrary to the emergence of AIDS and the first HIV preventive policies in the 1980s, it does not appear that either the onset of the ART era in 1996 or the new preventive strategies based on the use of antiretroviral drugs (such as *Treatment as Prevention* and *Pre-Exposure Prophylaxis)* in the late 2000s have modified old trends or generated new ones in the sexual practices studied here. If anything, they might rather have sustained emerging trends that had started earlier.

This longitudinal analysis also revealed an effect of age different from that usually seen in transversal analyses. In particular, we found that the frequency of sexual practices may increase lifelong, even at older ages.

Finally, similar trends observed in every generation from 1985 to 2011 may be interpreted in terms of strong period effects and sexual behaviours mostly influenced by contextual factors. On the one hand, past experience of some generations, such as those profoundly touched by AIDS in the 1980s and early 1990s, appeared not to affect their subsequent sexual practices differentially. On the other hand, the sexual practices of the younger generation showed no especially strong features, except fewer male partners. For instance, they reported more frequent anal intercourse than the older generations at the same age, but this was true for each generation compared with the older ones.

This analysis of large nationwide surveys showed that global trends in sexual behaviours may not necessarily respond to and indeed may precede changes in the epidemiological and preventive context. It underlined how difficult it can be for preventive policies to deal with the social dimensions of sexuality. A more detailed analysis of preventive practices of MSM will provide further information on these itineraries.
